# *SEPT6*_*TRIM33* Gene Fusion and Mutated TP53 Pathway Associate With Unfavorable Prognosis in Patients With B-Cell Lymphomas

**DOI:** 10.3389/fonc.2021.765544

**Published:** 2021-12-01

**Authors:** Haiying Fu, Huarong Zhou, Yanyan Qiu, Jianfei Wang, Zhiming Ma, Hongping Li, Feng Zhang, Chenxi Qiu, Jianzhen Shen, Tingbo Liu

**Affiliations:** ^1^ Department of Hematology, The Third Affiliated People’s Hospital of Fujian University of Traditional Chinese Medicine, The Third People’s Hospital of Fujian Province, Fuzhou, China; ^2^ Department of Hematology, Fujian Medical University Union Hospital, Fujian Institute of Hematology, Fujian Provincial Key Laboratory on Hematology, Fuzhou, China; ^3^ Research and Development Division, Oriomics Biotech Inc, Hangzhou, China

**Keywords:** B-cell lymphoma, gene fusion, mutated pathway, TP53 signaling pathway, Hippo pathway, prognosis

## Abstract

**Background:**

Mounting studies have sought to identify novel mutation biomarkers having diagnostic and prognostic potentials. Nevertheless, the understanding of the mutated pathways related to development and prognosis of B-cell lymphoma is still lacking. We aimed to comprehensively analyze the mutation alterations in genes of canonical signaling pathways and their impacts on the clinic outcomes of patients with B-cell lymphoma.

**Methods:**

Circulating cell-free DNA (cfDNA) samples from 79 patients with B-cell lymphomas were used for targeted sequencing with a 560-gene panel for depicting mutation landscapes and identifying gene fusion events. Gene ontology (GO) and Kyoto Encyclopedia of Genes and Genomes (KEGG) functional enrichment analyses of mutated genes were performed. The associations of mutation status of genes and seven canonical oncogenic pathways with progression-free survival (PFS) were assessed using Kaplan-Meier test and multivariate Cox analysis. The variant allele frequencies (VAFs) of genes in TP53 and Hippo pathways in paired baseline and post-treatment samples from 18 B-cell lymphoma patients were compared. Finally, the associations of identified fusion genes, mutated genes, and pathways with treatment response were evaluated based on objective response rates (ORRs) comparisons of groups.

**Results:**

We identified 666 mutations from 262 genes in baseline cfDNAs from 79 B-cell lymphoma patients, and found some genes were preferentially mutated in our cohort such as *GNAQ*, *GNAS*, *H3F3A*, *DNMT3A*, *HLA-A*, and *HLA-B*. These frequently mutated genes were significantly associated with negative “regulation of gene expression, epigenetic” and virus infections such as cytomegalovirus, Epstein-Barr virus, human immunodeficiency virus 1 infections. We detected five fusion genes in at least two patients with B-cell lymphoma, and among them, *TCF7L2*_*WT1* gene fusion was most frequently detected in 30.4% of patients (24 of 79 cases). *SEPT6*_*TRIM33* gene fusion, mutated TP53 and Hippo pathways were significantly associated with poor PFS, and *SEPT6*_*TRIM33* fusion gene and mutated TP53 pathway were independent prognostic factors for B-cell lymphoma. A decreased VAF of *TP53* p.Y88C and *LATS2* p.F972L was detected in patients with complete response to treatments. Moreover, a significant difference in ORR was observed in patients with NPM1_NR4A3 and SEPT6_TRIM33 fusions.

**Conclusions:**

*SEPT6*_*TRIM33* gene fusion and mutated TP53 and Hippo pathways may serve as prognostic makers for B-cell lymphoma patients.

## Introduction

B-cell lymphomas are heterogeneous malignancies that vary in clinical presentations and molecular phenotypes (1, 2), ranging from highly aggressive to very indolent. The complex pathogeneses of lymphomas result into various types of therapies, different treatment responses, and extremely variable clinical outcomes (3). B-cell lymphomas represent the most common subgroup of Non-Hodgkin lymphoma (NHL), which is the 13th most common cancer and the 12th most common cause of cancer death worldwide with over 544,352 new cases estimated for 2020 (4), thus posing a significant public health concern. In clinics, it is of great importance to assess the response to treatments and monitor clinical outcomes, which may affect the decision making in the wide range of therapeutic selection. Although imaging scans, including computed tomography (CT) and positron emission tomography (PET), are the traditional standards for initial diagnosis and early indication of treatment response of lymphoma, they are limited to use after onset of symptoms (5). With the development of next-generation sequencing, genomic analysis of circulating tumor DNA (ctDNA) emerges as an effective non-invasive method for identifying prognostic and predictive molecular makers in a broad spectrum of B-cell lymphomas using targeted sequencing panels (6).

Recently, mounting evidence has sought to identify novel molecular biomarkers with prognostic value and therapeutic response potentials in B-cell lymphomas. For example, the presence of MYC-IG rearrangement was identified as a predictor for treatment failure in patients with diffuse large B-cell lymphoma (DLBCL) who received immunochemotherapy (7). Novel evidence showed that DLBCL patients with MYC rearrangement, especially MYC double-hit or triple-hit constellation, had inferior outcomes (8). In addition, the occurrence of gene mutations like *CREBBP* and *EP300* mutations during disease course may predict worse overall survival (OS) and progression-free survival (PFS) for germinal center B cell-like DLBCL (9). *NFKBIE* functions as a NF-κB inhibitor, and the deletion of which was associated with inferior survival in primary mediastinal B-cell lymphoma (10). *KMD2D* mutation was associated with poor outcomes as well in patients with mantle cell lymphoma (MCL) (11). Nevertheless, these findings are only to assess the prognostic significance from the perspective of genomic alterations, and the comprehensive knowledge on the deregulated pathways caused by multi-mutations in B-cell lymphoma has rarely been addressed.

B-cell development and activation are accompanied by a series of genetic events, and abnormalities in these genetic events are responsible for the alterations of genes associated with B-cell proliferation or apoptosis, and consequently contribute to B lymphomagenesis (12). Usually, hallmarks of cancers include genetic alterations of gene fusions caused by chromosomal rearrangements or instability and genetic alterations in a group of genes in canonical signaling pathways including cell cycle, Hippo, Notch, PI3-Kinase, RTK-RAS, p53, and Wnt pathways (13, 14). However, the clinical implications of gene fusion events and mutated canonical pathways in B-cell lymphoma prognosis have not yet been fully elucidated. In this study, we sought to gain a global view of somatic mutations and gene fusions events, identify mutated pathways, as well as evaluate their impacts on the clinic outcomes of patients with B-cell lymphomas.

## Materials and Methods

### Study Population

Seventy-nine patients who were diagnosed as having B-cell lymphomas in the Union Hospital Affiliated to Fujian Medical University between December 2017 and April 2021 were included in this study. Pathological diagnosis was performed by pathological reviews to confirm subtypes of lymphomas following the criteria of WHO classification of Tumors of Hematopoietic and Lymphoid tissue criteria (15). Demographic and clinicopathologic characteristics and outcomes were collected. Peripheral blood (10 ml) was drawn from each patient for circulating cell-free DNA (cfDNA) profiling assays. The study design was approved by the Ethics Committee of the Union Hospital Affiliated to Fujian Medical University in accordance with the Declaration of Helsinki. All patients provided written informed consent.

### Targeted Panel Sequencing

Total DNAs from blood samples were extracted using the Magnetic Serum/Plasma DNA Maxi Kit (DP710, TIANGEN Biotechnology, China), following the manufacturer’s instructions. The extracted cfDNA samples were quantified using Qubit dsDNA HS Assay Kit (Invitrogen) and sheared into 150 to 200 bp fragments, and cfDNA with input concentrations (≥5 ng) and without overly genomic DNA contamination was subjected to library construction prior to fragment length analysis using Qsep100 (Bioptic, Taiwan, China). Fragmented DNA libraries were constructed using nano DNA library prep kit (for Illumina^®^) (Nanodigmbio, Nanjing, China) for end-repairing, adding A-tailing, and adapter ligation. DNA libraries were then subjected to PCR amplification and purification. The fragment quantification was performed using Qsep100 automatic nucleic acid analysis system (BiOptic, Taiwan, China). Subsequently, the lymphoma panel libraries were captured hybridly with a designed panel of 560 cancer-related genes (Pancare^®^, Oriomics Biotech Inc, Zhejiang, China) using xGen^®^ Hybridization and Wash Kit (IDT). The barcoded duplex adapters were eventually blocked by NadPrep NanoBlockers (for illumine) (Nanodigmbio, Nanjing, China). The quality control of final libraries was assessed by Qubit 3.0 fluorimeter (Invitrogen) and Qsep100 (Bioptic, Taiwan, China). The qualified libraries with minimum 10 ng/μl concentration were sequenced on the Illumina NextSeq 550 Dx platform with a mean coverage depth of 9,960× for captured regions.

### Bioinformatics Analysis

After obtaining the raw sequencing data in the Fastq format, the fastp 0.20.0 software was utilized to perform quality analysis by removing adapter sequence, reads containing N base calls, and low-quality reads (quality reading below 20) (16). All clean paired-end reads were aligned to the human reference genome (17) using Burrows-Wheeler Aligner (BWA). Variant calling was performed using Mutect2 from Genome Analysis Toolkit (GATK 3.4.0, https://software.broadinstitute.org/gatk/; Broad Institute, Cambridge, MA, USA). Then, the detected mutations were further selected following established criteria: (1) variant allele frequency (VAF) more than 0.5% with at least three minimum variant supporting reads; (2) dual UMI ≥1; (3) strand specificity ranging from 0.1 to 0.9; (4) EXAC_EAS or genomAD_EAS less than 0.05. Final mutations were annotated using ANNOVAR (18), and only functional mutations, e.g., non-synonmous, frameshift deletion and insertion were selected for subsequent bioinformatics analysis. DNAs from leukocytes of the same patients were sequenced as germline controls. Maftools package (https://bioconductor.org/packages/release/bioc/vignettes/maftools/) (19), an efficient tool for comprehensive analysis of somatic variant for cancers, was applied to analyze the number of variants in each sample and draw oncoplots for visualizing the mutation landscape. For fusion gene analysis, GeneFuse software (version 0.6.1) was used to detect fusions from fusion file including cancer-related fusions genes from COSMIC (17). In order to investigate the potential biological roles of mutated genes, we used ClusterProfiler in R software to conduct the Gene ontology (GO) and Kyoto Encyclopedia of Genes and Genomes (KEGG) functional analyses (12). The significant enrichment terms were identified by the threshold value of false discovery rate (FDR) <0.05. For mutated pathway analysis, one pathway was defined as mutated signaling pathway when patients harbored one or more mutated gene members of this pathway.

### Statistical Analysis

Statistical analyses were conducted using R package (version 3.61). PFS was defined as time elapsed between first sampling to disease progression, or death, or last follow-up, whichever came first. Kaplan-Meier test in survival package (version 3.1-11) was applied to assess associations of mutation status of genes and pathways with PFS. Factors significantly associated with PFS in the univariate analysis were subjected to multivariable Cox proportional hazards regression analysis. Objective response rate (ORR) was defined as the percentage of patients who received complete response (CR) or partial response (PR) during the treatment using response evaluation criteria in lymphoma (RECIL) (20). ORRs between groups were compared to explore the associations of fusion genes, mutated genes, and pathways with treatment response using Chi-square test. A *P* value of < 0.05 was considered statistically significant.

## Results

### Clinical Characteristics

The demographics and baseline characteristics of 79 B-cell lymphoma patients are presented in **Table 1**. The median age of patients at blood drawn was 56 years, and the numbers of males and females were comparable (40/39). Histologically, 55 (69.6%) patients were diagnosed with DLBCL, six (7.6%) with follicular lymphoma (FL), four (5.1%) with marginal zone lymphoma (MCL), and two (2.5%) with high-grade B-cell lymphoma (HBL). In addition to three cases without detailed subtype information, the other 76 patients comprised 85.5% aggressive B-cell lymphoma (N=65) and 14.5% indolent B-cell lymphoma (N=11). Among 72 cases with definite staging, 5 (6.3%), 14 (17.7%), 10 (12.7%), and 43 (54.4%) patients had stage I, II, III, IV lymphomas, respectively. Majorities of the patients received R-CHOP (34.2%) or R-COEP (27.8%) as their first-line therapy. Fifty-seven percentage of patients achieved CR (N=26) or PR (N=19) to treatments they received, while 8.9% of patient had a stable (SD, N=2) or progressive disease (PD, N=5), during a median follow-up of 7.08 months.

**Table 1:** Patient demographic and clinical characteristics (N=79).

Variables	N (%)
**Gender**	
Male	41 (51.9%)
Female	38 (48.1%)
**Age, years, median (interquartile range)**	56 (47-65)
**BMI (kg/m^2^)**	
18.5~23.9	45 (57.0%)
<18.5	8 (10.1%)
≥24	24 (30.4%)
Unknown	2 (2.5%)
**Smoking history**	
Yes	18 (22.8%)
No	56 (70.9%)
Unknown	5 (6.3%)
**Drinking history**	
Yes	26 (32.9%)
No	52 (65.8%)
Unknown	1 (1.3%)
**Pathological diagnosis**	
Diffuse large B-cell lymphoma	55 (69.6%)
Follicular lymphoma	6 (7.6%)
Marginal zone lymphoma	4 (5.1%)
High-grade B-cell lymphoma	2 (2.5%)
Other*	9 (11.4%)
Unknown	3 (3.8%)
**Stage**	
I	5 (6.3%)
II	14 (17.7%)
III	10 (12.7%)
IV	43 (54.4%)
Unknown	7 (8.9%)
**First-line therapy**	
R-CHOP	27 (34.2%)
CHOP	3 (3.8%)
R-miniCHOP	4 (5.1%)
R-COEP	22 (27.8%)
R-DA-EPOCH	3 (3.8%)
Other^#^	20 (25.3%)
**International Prognostic Index**	
0–1	27 (34.2%)
2	19 (24.1%)
3	15 (19.0%)
	11 (13.9%)
Unknown	7 (8.9%)
**Response to treatment**	
Complete response	26 (32.9%)
Partial response	19 (24.1%)
Stable disease	2 (2.5%)
Progressive disease	5 (6.3%)
Unknown	27 (34.2%)

BMI, body mass index; R-CHOP, rituximab plus cyclophosphamide, doxorubicin, vincristine, and prednisone; R-miniCHOP, rituximab with low-does CHOP chemotherapy regimen; R-COEP, rituximab plus cyclophosphamide, vincristine, etoposide, and prednisone; R-DA-EPOCH, rituximab with dose-adjusted etoposide, prednisone, vincristine, cyclophosphamide, and doxorubicin.

*Other includes subtypes of mantle cell lymphoma (MCL), marginal zone lymphoma (MZL), B lymphoblastic lymphoma, large B cell lymphoma, lymphoblastic lymphoma, Burkitt’s lymphoma, lymphoplasmacytic lymphoma, and primary central nervous system large B-cell lymphoma.

^#^Other includes regimens of cyclophosphamide, epirubicin, vincristine, and prednisone (CEOP), rituximab combined with lenalidomide (R2)-CEOP (R2-CEOP), R2-gemox, rituximab combined with methotrexate, cytarabine and dexamethasone (R-MAD), hyperfractionated cyclophosphamide, vincristine, doxorubicin, and dexamethasone (Hyper-CVAD), rituximab plus methotrexate (R-MTX), R2-CHOP and R2-COEP.

### Genomic Alterations in Patients With B-Cell Lymphomas

**Figure 1:**
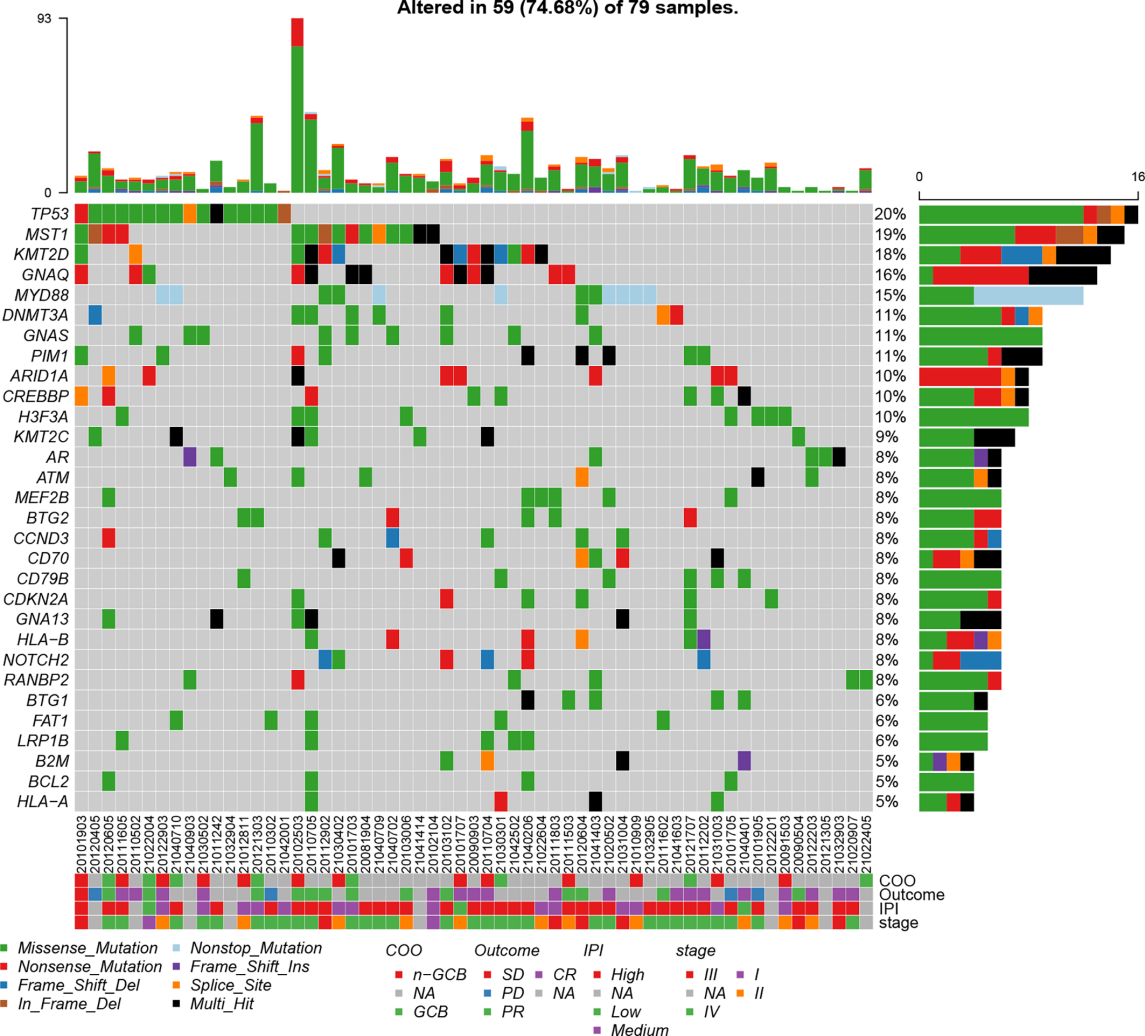
Mutation plot showing top 30 frequently mutated genes in 79 B-cell lymphoma patients. Color-coded is indicated by type of mutation. Y axis shows the percentages of patients with at least one mutation in the specified gene.

In order to gain a global view of the genomic variations of B-cell lymphomas, the somatic mutations and gene fusion events in 79 patients with B-cell lymphomas were identified based on targeted panel sequencing. In total, we identified 666 mutations, in which the major mutation types included missense mutation (79.0%), non-sense mutation (10.4%), frameshift deletion (5.1%), in-frameshift deletion (2.5%), frameshift insertion (2.4%), and non-stop mutation (0.6%). In addition, mutations from 262 of 560 cancer-related genes were detected in 59 patients, and among these mutated genes, 46 genes had a mutation frequency of more than 5%. The top 30 commonly mutated genes are shown in **Figure 1**, and the top 11 preference genes were *TP53* (20%), *MST1* (19%), *KMT2D* (18%), *GNAQ* (16%), *MYD88* (15%), *DNMT3A* (11%), *GNAS* (11%), *PIM1* (11%), *ARID1A* (10%), *CREBBP* (10%), and *H3F3A* (10%). Of note, there were nine preference mutated genes (e.g., *TP53*, *KMT2D*, *CREBBP*, *BCL2*, *ATM*, and *PIM1*) that were overlapped between the top 30 frequently mutated genes of our results, and the top 20 frequently mutated genes detected in lymphoid tissues of 413 DLBCL patients from COSMIC database. It also showed that the frequencies of these overlapped genes were comparable (**Supplementary Table 1**), demonstrating the reliability of our sequencing assays.

Gene fusions are an important class of somatic alterations in hematological malignancies (21). Thus, we examined the gene fusion events in our cohort and found that five fusion genes were detected in at least of two cases, comprising of *FAM65B*_*NTRK3*, *NPM1*_*NR4A3*, *RANBP2*_*MCPH1*, *SEPT6*_*TRIM33*, and *TCF7L2*_*WT1*. There were four patients having *SEPT6*_*TRIM33* gene fusion, while nine patients harbored *RANBP2*_*MCPH1* gene fusion. Notably, *TCF7L2*_*WT1* gene fusion was most frequently detected in 29.1% patients (23 of 79 cases) (**Table 2**).

**Table 2:** Fusion genes identified from79 B-cell lymphomas.

Fusion genes	Types	Detected patients
FAM65B_NTRK3	Intron of FAM65B(−):46Kb after exon 1|Intron of NTRK3(−):20Kb before exon 13	Pt58
	Intron of FAM65B(−):1Kb after exon 22|Intron of NTRK3(−):7Kb after exon 13	Pt37
NPM1_NR4A3	NPM1:exon3-NR4A3:intron7	Pt9, Pt10, Pt17
RANBP2_MCPH1	RANBP2:exon21-MCPH1:intron12	Pt4, Pt5, Pt6, Pt7, Pt9, Pt11, Pt14, Pt17, Pt18
SEPT6_TRIM33	SEPT6:intron2-TRIM33:intron1	Pt2, Pt5, Pt7, Pt16
TCF7L2_WT1	Intron of TCF7L2(+):24Kb before exon 5|Intron of WT1(−):39bp after exon 1	Pt38, Pt39, Pt69
	Intron of TCF7L2(+):24Kb before exon 5|Intron of WT1(−):74bp after exon 1	Pt1, Pt20, Pt22, Pt23, Pt29, Pt31, Pt48, Pt51, Pt54, Pt56, Pt57, Pt60, Pt67, Pt71, Pt72
	Intron of TCF7L2(+):24Kb before exon 6|Intron of WT1(−):2Kb before exon 2	Pt76
	TCF7L2:intron5-WT1:intron1	Pt2, Pt5, Pt7, Pt13

### Functional Enrichment Analysis of Somatic Mutated Genes

**Figure 2:**
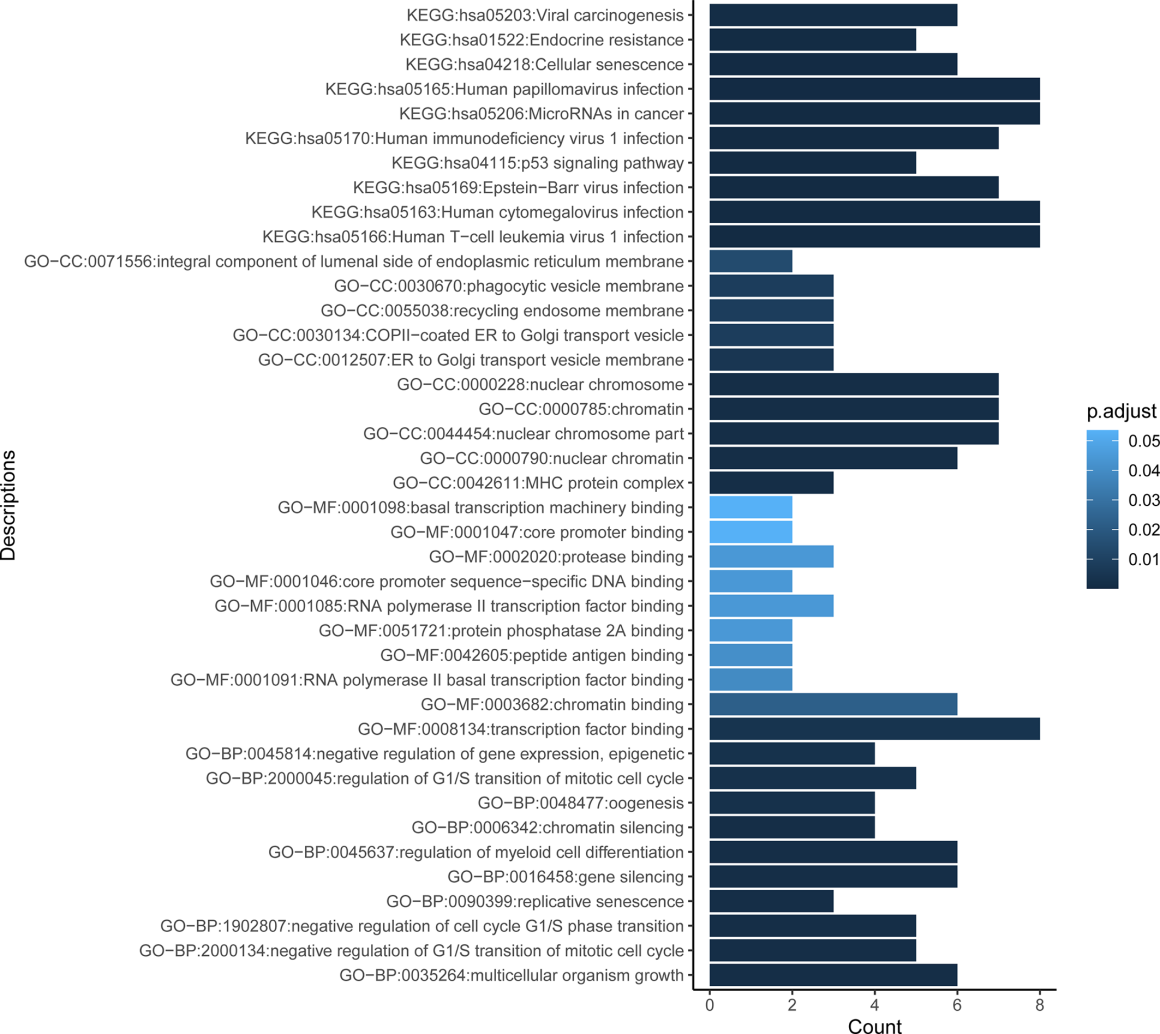
Functional enrichment result reveals the top 10 function terms for GO and KEGG analysis. Gene Ontology (GO) comprises three categories: molecular function (MF), biological process (BP), and cellular component (CC). X axis represents the count of genes enriched in corresponding terms. Color depth of columns is positively related with the P value of term. KEGG, Kyoto Encyclopedia of Genes and Genomes.

To better understand the biological roles of mutated genes, GO and KEGG enrichment analysis were performed. GO enrichment analysis revealed that the mutated genes were significantly enriched in regulation of G1/S transition of mitotic cell cycle (biological process [BP], e.g., CDKN2A, *ATM, TP53* and *BCL2*), gene silencing (BP, e.g., *DNMT3A*, *RANBP2*, and *KMT2D*), negative regulation of gene expression, epigenetic (BP, e.g., *H3F3A* and *DNMT3A*), MHC protein complex (cellular component [CC], *HLA-A*, *HLA-B*, and *B2M*), and transcription factor binding (molecular function [MF], e.g., *ARID1A*, *PIM1*, *AR*, and *CREBBP*). KEGG pathway analysis showed that the mutated genes were significantly associated with virus infections involved pathways such as human T-cell leukemia virus 1 infection (e.g., *HLA-A* and *HLA-B*), cytomegalovirus infection (e.g., *HLA-A, HLA-B, GNAQ, GNAS*, and *GNA13*), Epstein-Barr virus (EBV) infection (e.g., *HLA-A* and *HLA-B*), human immunodeficiency virus 1 (HIV) infection (e.g., *HLA-A, HLA-B*, and *GNAQ*), and papillomavirus infection (e.g., *HLA-A, HLA-B*, and *GNAS*) (**Figure 2**).

### Mutations in Seven Canonical Oncogenic Signaling Pathways

**Figure 3:**
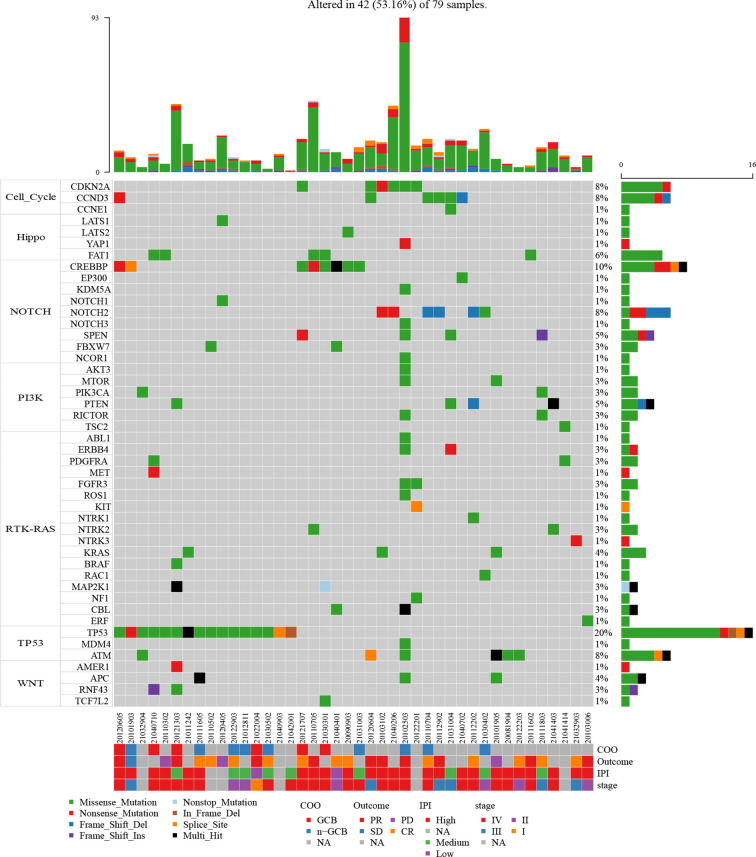
Mutation plot of seven canonical oncogenic signaling pathways (cell cycle, Hippo, Notch, PI3-Kinase, RTK-RAS, p53, and Wnt pathways) in 79 B-cell lymphoma patients. Color-coded is indicated by type of mutation. Y axis shows the percentages of patients with at least one mutation in the specified gene.

With the aim of identifying preferentially mutated pathways in B-cell lymphoma, we evaluated the mutational events of component genes involved in seven canonical oncogenic pathways. The mutation profiles of these pathways are shown in **Figure 3**. TP53 mutational profile was featured primarily by *TP5*3 mutations, while *CDKN2A*, *CCND3*, *FAT1*, *CREBBP*, and *PTEN* were the most frequently mutated genes in cell cycle, Hippo, NOTCH, and PI3K mutational profiles, respectively. Strikingly, TP53 and NOTCH pathways were both most commonly mutated oncogenic pathways in B-cell lymphoma in our cohort. Almost one-fifth of patients harbored mutations in component genes of each of these two pathways (24.05%, 19 of 79 cases). In addition, we found that 7 (8.86%), 6 (7.59%), and 6 (7.59%) of 79 patients had mutations in Hippo, WNT, and cell cycle pathways, respectively (**Supplementary Figure 1**). There were 19 and 21 patients harboring mutations in component genes of at least two mutated pathways and only one mutated pathway, respectively.

### Prognostic Roles of Fusion Genes, Mutated Genes, and Pathways

**Figure 4:**
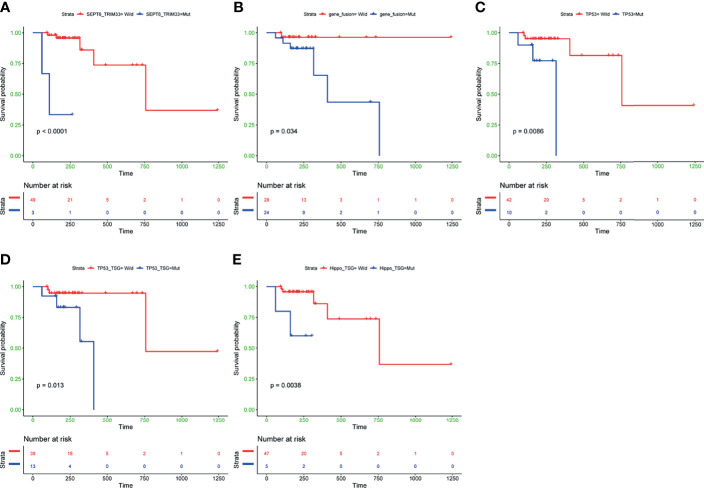
Kaplan-Meier curves of PFS differences between subgroups with and without *SEPT6*_*TRIM33* gene fusion **(A)**, any of five fusion genes detected in at least of two cases **(B)**, TP53 gene mutation **(C)**, mutated TP53 pathway **(D)**, mutated Hippo pathway **(E)**. PFS, progression-free survival. P values were calculated by the log-rank test.

To further investigate the clinical influence of genomic aberrations in groups of fusion genes, the top 30 commonly mutated genes, and seven canonical oncogenic pathways, we assessed the associations between the status of these genomic alterations and PFS in 52 patients with complete data. The baseline characteristics of these 52 patients are shown in **Supplementary Table 2**. The results revealed that among the five fusion genes, only patients with *SEPT6*_*TRIM33* fusion gene had significantly shorter PFS (*P*<0.0001, **Figure 4A**). In addition, patients harboring any of these five fusion genes showed a trend towards worse prognosis (*P*=0.034, **Figure 4B**). Unfavorable outcome was also observed in B-cell lymphoma patients (N=52) with mutated *TP53* gene (*P*=0.0086, **Figure 4C**), mutated TP53 pathway (*P*=0.013, **Figure 4D**), and mutated Hippo pathway (*P*=0.0038, **Figure 4E**). There was no significant difference in PFS between patients with and without mutations in other commonly mutated genes or pathways. We further conducted subgroup analysis in DLBCL patients (N=38), and the results showed that *TP53*-mutated DLBCL patients had a significantly unfavorable prognosis compared to *TP53* wild-type DLBCL patients (*P*=0.0047, **Supplementary Figure 2A**). Similarly, DLBCL patients without mutated TP53 pathway tended to have prolonged PFS (*P*=0.061, **Supplementary Figure 2B** **)**. However, we could not compare prognosis between DLBCL patients with and without mutated *SEPT6*_*TRIM33* fusion gene or Hippo pathway, due to the small number of patients having this genomic alteration.

To explore whether co-mutations of TP53 and Hippo pathways could have more significant impact than single TP53 or Hippo pathways mutations on the patients’ survival, we compared survivals across subgroups with co-mutations, with any mutations, and without a mutation in these two pathways. The results revealed that B-cell lymphoma patients with both wild-type TP53 and Hippo pathways had the best prognosis, while the patients with the presence of co-mutated TP53 and Hippo pathways had the worst PFS (*P*<0.0001, **Supplementary Figure 3**).

**Figure 5:**
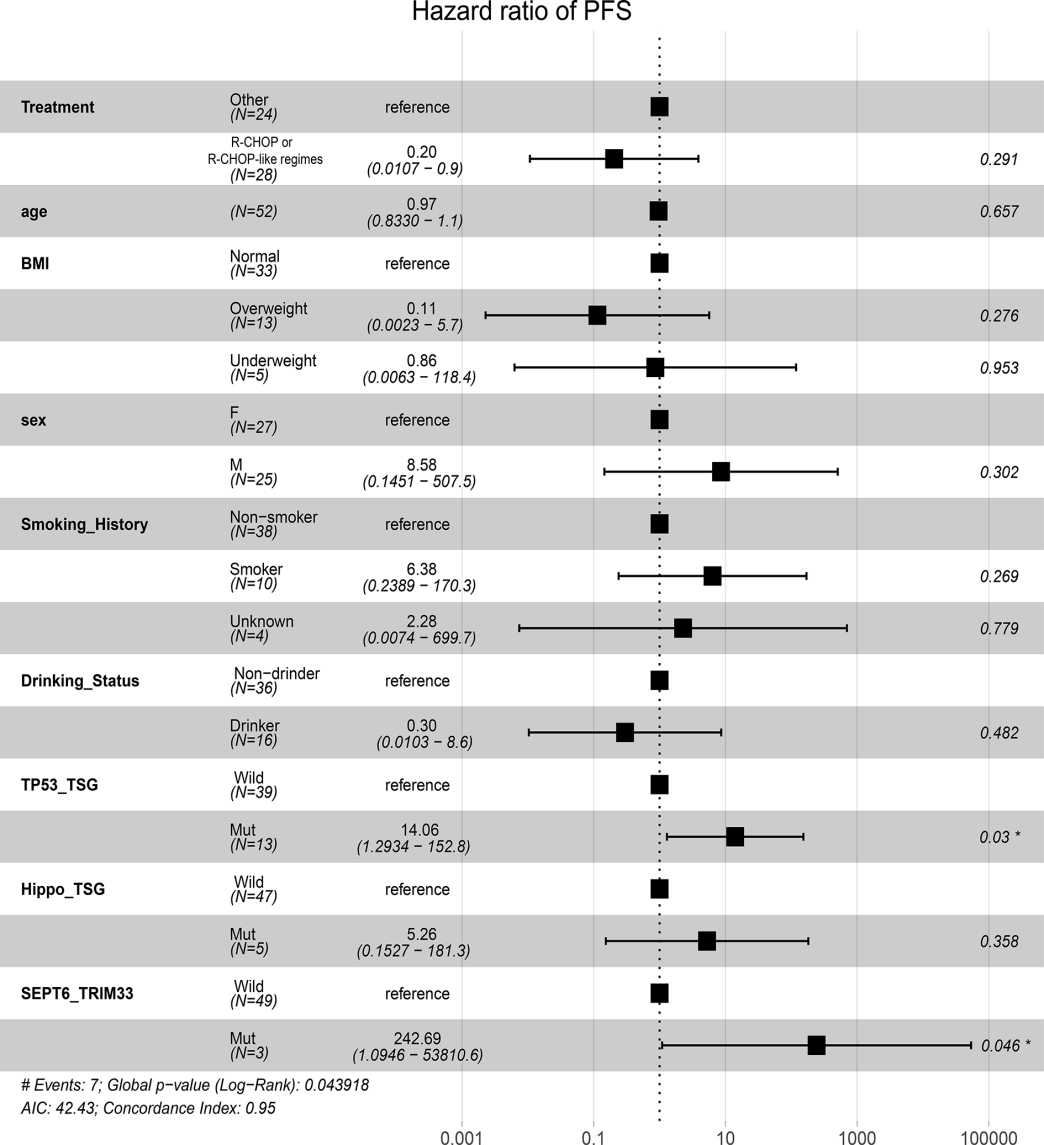
Prognostic significances of fusion gene, mutated pathways, and clinic factors by multivariable Cox proportional hazards regression analysis.

After identification of *SEPT6*_*TRIM33* fusion genes, mutated TP53 and Hippo pathways as factors significantly associated with prognosis of B-cell lymphomas, multivariable Cox analyses adjusted by clinic covariates [age, gender, BMI, smoking history, drinking status and treatment (R-CHOP or R-CHOP-like regimes *vs* others)] were conducted. The result suggested that *SEPT6*_*TRIM33* fusion gene [hazard ratio (HR): 242.69, *P*=0.046] and mutated TP53 pathway (HR: 14.06, *P*=0.03) were independent prognostic factors for B-cell lymphoma patients (**Figure 5**).

### VAFs Changes of Mutated Genes in TP53 and Hippo Pathways

**Figure 6:**
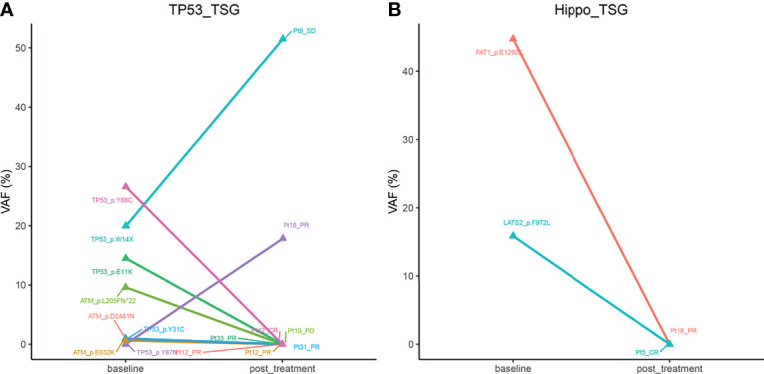
The alterations of mean VAFs of mutated genes in Tp53 **(A)** and hippo pathways **(B)** in paired baseline and post-treatment samples from 18 B-cell lymphoma patients. VAF, variant allele frequency.

Owing to our above findings that mutated TP53 and Hippo pathways were significantly associated with patient prognosis, we wondered whether the status of the genes in these two pathways may be altered after chemotherapy, which may be useful for guiding subsequent therapies. To that end, we analyzed the VAFs of genes in the TP53 and Hippo pathways in paired baseline and post-treatment samples from 18 B-cell lymphoma patients. The characteristics of these 18 patients are presented in **Supplementary Table 3**. As shown in **Figure 6**, Pt18 (DLBCL subtype), who achieved PR after four cycles of R-DA-EDOCH treatment, presented an increased VAF of *TP53* p.Y87N mutation (TP53 pathway; 0 *vs* 17.86%), but a decreased VAF of *FAT1* p.E1292K mutation (Hippo pathway; 44.74 *vs* 0%), which might be responsible for partial sensitivity or acquired resistance to R-DA-EDOCH. Interestingly, two CR patients detected a decreased VAF of *TP53 p.Y88C* (Pt42, DLBCL subtype, five cycles of R-miniCHOP) and LATS2 *p.F972L* (Pt5, FL subtype, four cycles of R-COEP), respectively. These results suggested that VAF changes of mutated genes in TP53 and Hippo pathways might be an indicator for treatment responses.

### Genomic Alterations Correlate to Treatment Response

In order to investigate the relationship between fusion genes, mutated genes and pathways, and response to treatment, ORRs were compared between patients with and without a specific genomic alteration. As a result, significant differences in ORR were observed for patients with NPM1_NR4A3 (33.33 *vs.* 88%, *P*=0.01) and SEPT6_TRIM33 (33.33 *vs.* 88%, *P*=0.01) fusions *versus* patients without corresponding fusion (**Table 3**). Although patients with other fusion genes, *TP53* mutation (70 *vs.* 88.37%, *P*=0.14), mutated TP53 (69 *vs.* 90%, *P*=0.07), and Hippo pathways (60 *vs.* 87.5%, *P*=0.33) trended towards a lower ORR, the difference was not statistically significant (**Table 3**).

**Table 3:** Relationship between genomic alterations and clinical response to treatment.

Genomic alterations	Group (n)	CR+PR	PD+SD	ORR (%)	P.value
NPM1_NR4A3	Mut (3)	1	2	33.33%	0.01
	Wild (50)	44	6	88.00%	
FAM65B_NTRK3	Mut (1)	0	1	0.00%	0.02
	Wild (52)	45	7	86.54%	
SEPT6_TRIM33	Mut (3)	1	2	33.33%	0.01
	Wild (50)	44	6	88.00%	
RANBP2_MCPH1	Mut (9)	7	2	77.78%	0.51
	Wild (44)	38	6	86.36%	
TCF7L2_WT1	Mut (14)	11	3	78.57%	0.44
	Wild (39)	34	5	87.18%	
TP53	Mut (10)	7	3	70.00%	0.14
	Wild (43)	38	5	88.37%	
TP53_TSG	Mut (13)	9	4	69.23%	0.07
	Wild (40)	36	4	90.00%	
Hippo_TSG	Mut (5)	3	2	60.00%	0.32
	Wild (48)	42	6	87.50%	

CR, complete response; PR, partial response; SD, stable disease; PD, progressive disease; ORR, objective response rate.

## Discussion

A handful of somatic mutations in B-cell lymphomas has been identified to participate in cancer development and progression (22, 23). Notably, the lymphomagenesis and progression caused by the coordination of multiple mutated genes at the pathway level are considered more reasonable than at the single gene level (24, 25). It is known that genomic alterations and their affected pathways may have diagnostic, prognostic, and therapeutic implications for B-cell lymphomas (2). However, the understanding of mutated pathways related to B-cell lymphoma development and patient prognosis is still inadequate, especially when genomic biomarkers are detected using the liquid biopsy approach. To that end, we detected the mutation landscapes and gene fusion events in cfDNA samples of 79 B-cell lymphoma patients based on targeted panel sequence to screen novel biomarkers having prognosis values. In this study, we found five gene fusions (e.g., *TCF7L2*_*WT1*) were frequently detected in B-cell lymphomas. The *SEPT6*_*TRIM33* gene fusion, mutated TP53, and Hippo pathways conferred unfavorable prognosis. Moreover, *SEPT6*_*TRIM33* fusion gene and mutated TP53 pathway were prognostic factors independent of clinical confounders. These findings may provide novel molecular biomarkers for prognostic stratification and potential therapeutic targets of B-cell lymphomas.

In this work, we totally identified 666 mutations from 262 genes in cfDNA samples from 79 B-cell lymphomas patients. Through comparing preferentially mutated genes between our cohort and 413 DLBCL tissues samples from COSMIC database, we found some genes were especially mutated with high frequencies in our cohort such as *GNAQ*, *GNAS*, *H3F3A*, *DNMT3A*, *HLA-A*, and *HLA-B*, in addition to nine overlapped genes (e.g., *TP53*, *KMT2D*, *MYD88*, *ATM*, and *BCL2*). The mutations identified in these overlapped genes are responsible for regulation of G1/S transition of mitotic cell cycle (e.g., *ATM* and *TP53*). Tumor suppressor-associated mutations in *TP53* and *ATM* may not induce cell cycle arrest and DNA repair (26). Notably, these frequently mutated genes in our cohort were significantly associated with functional categories involved negative regulation of gene expression, epigenetic (*H3F3A* and *DNMT3A*), and virus infections such as cytomegalovirus, EBV, HIV, and papillomavirus infections (e.g., *GNAS*, *GNAQ*, *HLA-A*, and *HLA-B*). *GNAS* and *GNAQ* encode G-protein alpha subunit, and the viruses like cytomegalovirus and EBV have been identified to encode G proteins and their coupled receptors in their genomes to induce cancer initiation (27). The variations of antigen-presentation molecules HLA may alter epitope recognition by the T-cell receptor, which enables HIV to escape cytotoxic T lymphocytes responses (28). Of note, NHL is one of the most common cancers in HIV-positive patients (29). Collectively, the present work gives insight into the biological processes and pathways involved in lymphomagenesis.

Fusion genes have been extensively used in cancer diagnosis and determining prognostic impact (30). Recently, adopting capture-based panel sequencing approach has enabled the identification of novel gene fusions in cancer patients (31). In this study, we identified novel *TCF7L2*_*WT1* gene fusion as one of the most frequently detected genomic aberrations in B-cell lymphomas. Transcription Factor 7-Like 2 (*TCF7L2*), also known as TCF4 transcription factor, is a key effector for the activation of Wnt signaling pathway in adult T cell leukemia cells (32). Wilms’ tumor 1 (WT1), a tumor-suppressor gene, can interact with lymphoid enhancer-binding factor 1 (LEF1) to mediate Wnt signaling activation (33). TCF4/LEF1 DNA binding effectors can interact with β-catenin to increase cyclin D1 and c-Myc expression, demonstrating the activation of Wnt signaling pathway (34). In line with the evidence that canonical Wnt signaling plays an important role in the development and progression of B-cell lymphomas (35), in our study population, we inferred the novel TCF7L2_WT1 gene fusion may associate with B-cell lymphomas progression through triggering the dysregulation of Wnt signaling. Although TCF7L2_WT1 gene fusion was not identified as a validated genomic alteration in tumors, an identified partner gene of *EWSR1* for *WT1* (WT1-EWSR1) reported in COSMIC database was also found as a partner gene of *TCF7L2* in colon adenocarcinom (36). In addition, our result revealed the prognostic value of *SEPT6*_*TRIM33* gene fusion in B-cell lymphomas. Septin 6 (*SEPT6*), a member of the septin family of GTPases, which preserves breakpoint at chromosomes Xq24, was found as a fusion partner with Leukemia gene (MLL1 or KMT2A) related to leukemia (37). Tripartite Motif Containing 33 (TRIM33), acting as an E3 ubiquitin-protein ligase, can prevent apoptosis in B lymphoblastic leukemia though mediating Bim activation (38). In addition, TRIM33 located at 1p13 region was identified as a tumor suppressor chronic myelomonocytic leukemia and associated with the survival of all B cell neoplasms (39). Combined with our survival results, we suggested that *SEPT6-TRIM33* gene fusion conferred poor prognosis in B cell neoplasms, which was valuable for prognostication and therapeutic decision making.

In this study, we analyzed the mutation profiles of seven canonical oncogenic pathways in B-cell lymphoma and found TP53 pathway was one of the most frequently mutated oncogenic pathways in B-cell lymphoma cohort. The regulation of TP53 governs a multitude of cellular processes, including cell cycle arrest, apoptosis, and changes in metabolism (40). Mutations in this pathway may cause genomic instability and deregulated transcription of genes involved in cell cycle, DNA repair, and apoptosis (41). Reportedly, somatic mutations in abnormal TP53 pathway and *TP53* itself are responsible for lymphoma generation, progression, and invasion (41). We detected mutations in TP53 pathway genes such as *MDM4* and *ATM*, especially *TP53*, which acts as a vital player in the cancer arena, was the most frequently mutated genes in B-cell lymphomas in our cohort (20%, 16/79). Mutations in p53 or its modulators such as MDM4 and ATM may affect p53 tumor suppressor function (42). In addition, we observed the mutations of *TP53* gene and mutated TP53 pathways were implicated with unfavorable patient prognosis. This was consistent with a previous study, in which *TP53* was mutated in 23.8% of aggressive B-cell lymphoma, and mutated *TP53* could serve as an adverse independent predictor for PFS (43).

Hippo signaling is another important tumor suppressor pathway, in which the core of module comprises of the tumor-suppressive MST-LATS kinases and downstream oncogenic effectors *YAP* and *TAZ* (44). So far, the mutations of genes in Hippo pathway have been rarely researched, possibly due to their low frequencies. Especially, DLBCL patients were reported to have few somatic alterations in Hippo pathway than other cancer types (45). In this study in B-cell lymphomas, we also found the low mutation frequencies of the major components of Hippo pathway like *LATS1* (1%), *LATS2* (1%), *YAP1* (1%), and *FAT1* (6%). Nonetheless, our result suggested the poor prognosis of mutated Hippo pathway for B-cell lymphoma. In addition, our result revealed that B-cell lymphoma patients with the presence of co-mutated TP53 and Hippo pathways had the worst prognosis, compared to patients with mutations in either pathway or without any mutation in both pathways. This result can be explained by that there may be a direct cross-talk between TP53 and Hippo tumor-suppressor pathways in multiple molecular interfaces such as p53-LATS axis (40) and p53-PTPN14-YAP axis (46).

Interestingly, a significantly lower ORR was observed in patients with *SEPT6-TRIM33* fusion, which was consistent with shorter PFS in these patients. Although patients with TP53 mutation, mutated TP53 and Hippo pathways trended towards a lower ORR, the difference was not statistically significant. Notably, PFS was significantly shorter in these patients. Aberrant activation of YAP/TAZ or loss-of-function of tumor suppressors in the Hippo pathway enhances tumor cell resistance against anticancer therapeutic drugs (47). *TP5*3 mutations are related to poor or no responses to chemotherapy such as cytarabine, rituximab, and autologous stem-cell transplant for mantle cell lymphoma (47). In our cohort, we found *TP53* p.Y87N mutation in Pt18 (DLBCL subtype) might induce resistance against DA-EDOCH. Integrating, these findings demonstrated that patients with specific mutation alterations, particularly in tumor-suppressor genes or pathways, might associate with poor treatment response and PFS. It has revealed that Verteporfin can reverse 5-Fu resistance in colorectal cancer through targeting the Hippo pathway in a YAP-dependent manner (48). Thus, identification of alterations in TP53 and Hippo pathways or novel gene fusions that are implicated in drug resistance and poor prognosis may guide pharmacological interventions by targeting the alterations and thus improve survival of B-cell lymphoma patients.

Despite this study has identified novel genomic biomarkers associated with prognosis of B-cell lymphoma patients, a major limitation of this study was a relatively small sample size, especially the small number of patients with identified fusion genes or mutated Hippo pathways, which makes subgroup analyses infeasible. Moreover, although we performed *in silico* analyses (GO and KEGG) to indicate the potential functional roles of mutated genes identified in this study, future *in vivo* and *in vitro* studies may further reveal the biological and molecular mechanisms underlying the associations between these genomic biomarkers and outcomes of B-cell lymphomas, especially DLBCL. Other limitations included heterogeneous patients with various therapeutic regimens, relatively short follow-up time, and the lack of important covariates such as education level, performance status. Therefore, larger prospective studies are needed to further verify our present findings.

In summary, we comprehensively analyzed the molecular profiling of B-cell lymphoma and found that *SEPT6*_*TRIM33* fusion gene, mutations in single *TP53* gene, mutated TP53 and Hippo pathways would together predict inferior prognosis for B-cell lymphomas. The present work also gives insight into the potential biological processes and pathways involved in lymphomagenesis. The study may provide novel genomic biomarkers of prognostic significance and potential therapeutic targets of B-cell lymphomas.

## Data Availability Statement

Data from this study, including the detected gene mutation points and fusion genes of each sample, are available in the main text, supplementary materials, or have been deposited in Figshare in the repository DOI: 10.6084/m9.figshare.16902844. Raw genetic sequencing data in this article are not readily available due to restrictions by national legislation, specifically the Administrative Regulations of the People’s Republic of China on Human Genetic Resources. Requests to acquire the sequencing data can be directed to the corresponding authors.

## Ethics Statement

The studies involving human participants were reviewed and approved by the Ethics Committee of the Union Hospital Affiliated to Fujian Medical University. The patients/participants provided their written informed consent to participate in this study.

## Author Contributions

HF, JS, and TL conceived and designed the project. HZ, YQ, and FZ organized sample collection. ZM and CQ analyzed and interpreted the data. HF, JS, TL, and JW discussed the results. HF wrote the manuscript. TL and HL edited the manuscript. All authors contributed to the article and approved the submitted version.

## Funding

We acknowledge the financial contribution by a grant from the Joints Funds for the Innovation of Science and Technology, Fujian Province (2018Y9010 and 2019Y9069), Construction project of Fujian medical center of hematology (Min201704), and National and Fujian Provincial Key Clinical Specialty Discipline Construction Program, P.R.C. [(2011)1018 and (2012)149].

## Conflict of Interest

JW, ZM, and HL are employees of Oriomics Biotech Inc.

The remaining authors declare that the research was conducted in the absence of any commercial or financial relationships that could be construed as a potential conflict of interest.

## Publisher’s Note

All claims expressed in this article are solely those of the authors and do not necessarily represent those of their affiliated organizations, or those of the publisher, the editors and the reviewers. Any product that may be evaluated in this article, or claim that may be made by its manufacturer, is not guaranteed or endorsed by the publisher.

## Supplementary Material

Supplementary Table 1 The overlapped mutated genes between the top 20 commonly mutated genes from COSMIC database and top 30 mutated genes our cohort.

Supplementary Table 2 Clinical characteristics of 52 patients for prognosis analysis.

Supplementary Table 3 Characteristics of subgroup patients with paired baseline and post-treatment samples (N=18).

Supplementary Figure 1 Pie chart shows the mutation rates of seven canonical oncogenic signaling pathways (cell cycle, Hippo, Notch, PI3-Kinase, RTK-RAS, p53, and Wnt pathways) in 79 patients.

Supplementary Figure 2 Kaplan-Meier curves of PFS differences between the *TP53*-mutated and *TP53* wild-type DLBCL patients **(A)** and between DLBCL patients with or without mutated TP53 pathway **(B)**.

Supplementary Figure 3 Kaplan-Meier curves of PFS differences across subgroups with co-mutations, any mutations, and without any mutation in TP53 and Hippo pathways.

## References

[1] Solé C Larrea E Di Pinto G Tellaetxe M Lawrie CH. MiRNAs in B-Cell Lymphoma: Molecular Mechanisms and Biomarker Potential. Cancer Lett (2017) 405:79–89. doi: 10.1016/j.canlet.2017.07.020 28757417

[2] Onaindia A Medeiros LJ Patel KP. Clinical Utility of Recently Identified Diagnostic, Prognostic, and Predictive Molecular Biomarkers in Mature B-Cell Neoplasms. Mod Pathol (2017) 30(10):1338–66. doi: 10.1038/modpathol.2017.58 28664939

[3] Rosenquist R Beà S Du MQ Nadel B Pan-Hammarström Q. Genetic Landscape and Deregulated Pathways in B-Cell Lymphoid Malignancies. J Intern Med (2017) 282(5):371–94. doi: 10.1111/joim.12633 28631441

[4] Sung H Ferlay J Siegel RL Laversanne M Soerjomataram I Jemal A. Global Cancer Statistics 2020: GLOBOCAN Estimates of Incidence and Mortality Worldwide for 36 Cancers in 185 Countries. CA Cancer J Clin (2021) 71(3):209–49. doi: 10.3322/caac.21660 33538338

[5] Melani C Wilson WH Roschewski M. Monitoring Clinical Outcomes in Aggressive B-Cell Lymphoma: From Imaging Studies to Circulating Tumor DNA. Best Pract Res Clin Haematol (2018) 31(3):285–92. doi: 10.1016/j.beha.2018.07.004 30213398

[6] Darrah JM Herrera AF. Updates on Circulating Tumor DNA Assessment in Lymphoma. Curr Hematol Malig Rep (2018) 13(5):348–55. doi: 10.1007/s11899-018-0468-4 30136210

[7] Copie-Bergman C Cuillière-Dartigues P Baia M Briere J Delarue R Canioni D. MYC-IG Rearrangements Are Negative Predictors of Survival in DLBCL Patients Treated With Immunochemotherapy: A GELA/LYSA Study. Blood (2015) 126(22):2466–74. doi: 10.1182/blood-2015-05-647602 26373676

[8] Rosenwald A Bens S Advani R Barrans S Copie-Bergman C Elsensohn MH. Prognostic Significance of MYC Rearrangement and Translocation Partner in Diffuse Large B-Cell Lymphoma: A Study by the Lunenburg Lymphoma Biomarker Consortium. J Clin Oncol (2019) 37(35):3359–68. doi: 10.1200/jco.19.00743 31498031

[9] Juskevicius D Jucker D Klingbiel D Mamot C Dirnhofer S Tzankov. a. Mutations of CREBBP and SOCS1 Are Independent Prognostic Factors in Diffuse Large B Cell Lymphoma: Mutational Analysis of the SAKK 38/07 Prospective Clinical Trial Cohort. J Hematol Oncol (2017) 10(1):1–10. doi: 10.1186/s13045-017-0438-7 28302137 PMC5356266

[10] Mansouri L Noerenberg D Young E Mylonas E Abdulla M Frick M. Frequent NFKBIE Deletions Are Associated With Poor Outcome in Primary Mediastinal B-Cell Lymphoma. Blood (2016) 128(23):2666–70. doi: 10.1182/blood-2016-03-704528 27670424

[11] Ferrero S Rossi D Rinaldi A Bruscaggin A Spina V Eskelund CW. KMT2D Mutations and TP53 Disruptions Are Poor Prognostic Biomarkers in Mantle Cell Lymphoma Receiving High-Dose Therapy: A FIL Study. Haematologica (2020) 105(6):1604–12. doi: 10.3324/haematol.2018.214056 PMC7271566 31537689

[12] Meng X Min Q Wang JY. B Cell Lymphoma. Adv Exp Med Biol (2020) 1254:161–81. doi: 10.1007/978-981-15-3532-1_12 32323276

[13] López-Nieva P Fernández-Navarro P Graña-Castro O Andrés-León E Santos J Villa-Morales M. Detection of Novel Fusion-Transcripts by RNA-Seq in T-Cell Lymphoblastic Lymphoma. Sci Rep (2019) 9(1):5179. doi: 10.1038/s41598-019-41675-3 30914738 PMC6435891

[14] Sanchez-Vega F Mina M Armenia J Chatila WK Luna A La KC. Oncogenic Signaling Pathways in the Cancer Genome Atlas. Cell (2018) 173(2):321–37.e310. doi: 10.1016/j.cell.2018.03.035 29625050 PMC6070353

[15] Bruneau J Molina TJ. WHO Classification of Tumors of Hematopoietic and Lymphoid Tissues. In: Molina TJ, editor. Hematopathology. (2020). (Cham: Springer International Publishing (2020). p. 501–5.

[16] Chen S Zhou Y Chen Y Gu J. Fastp: An Ultra-Fast All-in-One FASTQ Preprocessor. Bioinformatics (2018) 34(17):i884–90. doi: 10.1093/bioinformatics/bty560 PMC6129281 30423086

[17] Chen S Liu M Huang T Liao W Xu M Gu J. Genefuse: Detection and Visualization of Target Gene Fusions From DNA Sequencing Data. Int J Biol Sci (2018) 14(8):843–8. doi: 10.7150/ijbs.24626 PMC6036752 29989075

[18] Wang K Li M Hakonarson H. ANNOVAR: Functional Annotation of Genetic Variants From High-Throughput Sequencing Data. Nucleic Acids Res (2010) 38(16):e164–4. doi: 10.1093/nar/gkq603 PMC2938201 20601685

[19] Mayakonda A Lin DC Assenov Y Plass C Koeffler HP. Maftools: Efficient and Comprehensive Analysis of Somatic Variants in Cancer. Genome Res (2018) 28(11):1747–56. doi: 10.1101/gr.239244.118 PMC6211645 30341162

[20] Younes A Hilden P Coiffier B Hagenbeek A Salles G Wilson W. International Working Group Consensus Response Evaluation Criteria in Lymphoma (RECIL 2017). Ann Oncol (2017) 28(7):1436–47. doi: 10.1093/annonc/mdx097 PMC5834038 28379322

[21] Lindqvist CM Nordlund J Ekman D Johansson A Moghadam BT Raine A. The Mutational Landscape in Pediatric Acute Lymphoblastic Leukemia Deciphered by Whole Genome Sequencing. Hum Mutat (2015) 36(1):118–28. doi: 10.1002/humu.22719 PMC4309499 25355294

[22] Mareschal S Pham-Ledard A Viailly PJ Dubois S Bertrand P Maingonnat C. Identification of Somatic Mutations in Primary Cutaneous Diffuse Large B-Cell Lymphoma, Leg Type by Massive Parallel Sequencing. J Invest Dermatol (2017) 137(9):1984–94. doi: 10.1016/j.jid.2017.04.010 28479318

[23] Bolen CR Klanova M Trneny M Sehn LH He J Tong J. Prognostic Impact of Somatic Mutations in Diffuse Large B-Cell Lymphoma and Relationship to Cell-of-Origin: Data From the Phase III GOYA Study. Haematologica (2020) 105(9):2298–307. doi: 10.3324/haematol.2019.227892 PMC7556630 33054054

[24] Zhang W Wang SL. An Integrated Framework for Identifying Mutated Driver Pathway and Cancer Progression. IEEE/ACM Trans Comput Biol Bioinform (2019) 16(2):455–64. doi: 10.1109/tcbb.2017.2788016 29990286

[25] Vandin F Upfal E Raphael BJ. *De Novo* Discovery of Mutated Driver Pathways in Cancer. Genome Res (2012) 22(2):375–85. doi: 10.1101/gr.120477.111 PMC3266044 21653252

[26] Moia R Boggione P Mahmoud AM Kodipad AA Adhinaveni R Sagiraju S. Targeting P53 in Chronic Lymphocytic Leukemia. Expert Opin Ther Targets (2020) 24(12):1239–50. doi: 10.1080/14728222.2020.1832465 33016796

[27] Arang N Gutkind JS. G Protein-Coupled Receptors and Heterotrimeric G Proteins as Cancer Drivers. FEBS Lett (2020) 594(24):4201–32. doi: 10.1002/1873-3468.14017 PMC8849590 33270228

[28] Zimbwa P Milicic A Frater J Scriba TJ Willis A Goulder PJR. Precise Identification of a Human Immunodeficiency Virus Type 1 Antigen Processing Mutant. J Virol (2007) 81(4):2031–8. doi: 10.1128/JVI.00968-06 PMC1797578 17108020

[29] Zhang YX Gui XE Zhong YH Rong YP Yan YJ. Cancer in Cohort of HIV-Infected Population: Prevalence and Clinical Characteristics. J Cancer Res Clin Oncol (2011) 137(4):609–14. doi: 10.1007/s00432-010-0911-y PMC11827777 20532560

[30] Saleem M Yusoff NM. Fusion Genes in Malignant Neoplastic Disorders of Haematopoietic System. Hematology (2016) 21(9):501–12. doi: 10.1080/10245332.2015.1106816 26871368

[31] Wang D Ma K Deng W Li J Xiang S Zhang Y. Development and Analytical Validation of a Targeted Next-Generation Sequencing Panel to Detect Actionable Mutations for Targeted Therapy. Onco Targets Ther (2021) 14:2423–31. doi: 10.2147/OTT.S299381 PMC8039190 33854338

[32] Kurashina R Ohyashiki JH Kobayashi C Hamamura R Zhang Y Hirano T. Anti-Proliferative Activity of Heat Shock Protein (Hsp) 90 Inhibitors *via* β-catenin/TCF7L2 Pathway in Adult T Cell Leukemia Cells. Cancer Lett (2009) 284(1):62–70. doi: 10.1016/j.canlet.2009.04.012 19464103

[33] Tan H-Y Wang N Li S Hong M Guo W Man K. Repression of WT1-Mediated LEF1 Transcription by Mangiferin Governs β-Catenin-Independent Wnt Signalling Inactivation in Hepatocellular Carcinoma. Cell Physiol Biochem (2018) 47(5):1819–34. doi: 10.1159/000491063 29953980

[34] Mao CD Byers SW. Cell-Context Dependent TCF/LEF Expression and Function: Alternative Tales of Repression, De-Repression and Activation Potentials. Crit Rev Eukaryot Gene Expr (2011) 21(3):207–36. doi: 10.1615/critreveukargeneexpr.v21.i3.10 PMC3434703 22111711

[35] Janovská P Bryja V. Wnt Signalling Pathways in Chronic Lymphocytic Leukaemia and B-Cell Lymphomas. Br J Pharmacol (2017) 174(24):4701–15. doi: 10.1111/bph.13949 PMC5727250 28703283

[36] Krystel-Whittemore M Taylor MS Rivera M Lennerz JK Le LP Dias-Santagata D. Novel and Established EWSR1 Gene Fusions and Associations Identified by Next-Generation Sequencing and Fluorescence in-Situ Hybridization. Hum Pathol (2019) 93:65–73. doi: 10.1016/j.humpath.2019.08.006 31430493

[37] Peterson EA Petty EM. Conquering the Complex World of Human Septins: Implications for Health and Disease. Clin Genet (2010) 77(6):511–24. doi: 10.1111/j.1399-0004.2010.01392.x PMC5087971 20236126

[38] Wang E Kawaoka S Roe J-S Shi J Hohmann AF Xu Y. The Transcriptional Cofactor TRIM33 Prevents Apoptosis in B Lymphoblastic Leukemia by Deactivating a Single Enhancer. Elife (2015) 4:e06377. doi: 10.7554/eLife.06377 25919951 PMC4409649

[39] Crawford LJ Johnston CK Irvine AE. TRIM Proteins in Blood Cancers. J Cell Communication Signaling (2018) 12(1):21–9. doi: 10.1007/s12079-017-0423-5 PMC5842186 29110249

[40] Furth N Aylon Y Oren M. P53 Shades of Hippo. Cell Death Differ (2018) 25(1):81–92. doi: 10.1038/cdd.2017.163 28984872 PMC5729527

[41] Lu TX Young KH Xu W Li JY. TP53 Dysfunction in Diffuse Large B-Cell Lymphoma. Crit Rev Oncol Hematol (2016) 97:47–55. doi: 10.1016/j.critrevonc.2015.08.006 26315382

[42] Post SM Pant V Abbas H Quintás-Cardama A. Prognostic Impact of the MDM2SNP309 Allele in Leukemia and Lymphoma. Oncotarget (2010) 1(3):168. doi: 10.18632/oncotarget.123 21301048 PMC3157715

[43] Zenz T Kreuz M Fuge M Klapper W Horn H Staiger AM. TP53 Mutation and Survival in Aggressive B Cell Lymphoma. Int J Cancer (2017) 141(7):1381–8. doi: 10.1002/ijc.30838 28614910

[44] Gomez M Gomez V Hergovich A. The Hippo Pathway in Disease and Therapy: Cancer and Beyond. Clin Transl Med (2014) 3:22. doi: 10.1186/2001-1326-3-22 25097725 PMC4107774

[45] Wang Y Xu X Maglic D Dill MT Mojumdar K Ng PK-S. Comprehensive Molecular Characterization of the Hippo Signaling Pathway in Cancer. Cell Rep (2018) 25(5):1304–17. doi: 10.1016/j.celrep.2018.10.001 PMC6326181 30380420

[46] Aylon Y Oren M. Tumor Suppression by P53: Bring in the Hippo! Cancer Cell (2017) 32(4):397–9. doi: 10.1016/j.ccell.2017.09.010 29017051

[47] Eskelund CW Dahl C Hansen JW Westman M Kolstad A Pedersen LB. TP53 Mutations Identify Younger Mantle Cell Lymphoma Patients Who do Not Benefit From Intensive Chemoimmunotherapy. Blood (2017) 130(17):1903–10. doi: 10.1182/blood-2017-04-779736 28819011

[48] Huang C Chen Z Yang C Chen L Lai C Zhang Y. Combinational Inhibition of EGFR and YAP Reverses 5-Fu Resistance in Colorectal Cancer. J Cancer (2020) 11(18):5432–9. doi: 10.7150/jca.44775 PMC7391200 32742490

