# Supplementary figures and images for: SEPT6_TRIM33 Gene Fusion and Mutated TP53 Pathway Associate With Unfavorable Prognosis in Patients With B-Cell Lymphomas
Source: Front Oncol. 2021 Dec 1;11:765544. doi: 10.3389/fonc.2021.765544 (PMC8671703; doi:10.3389/fonc.2021.765544)

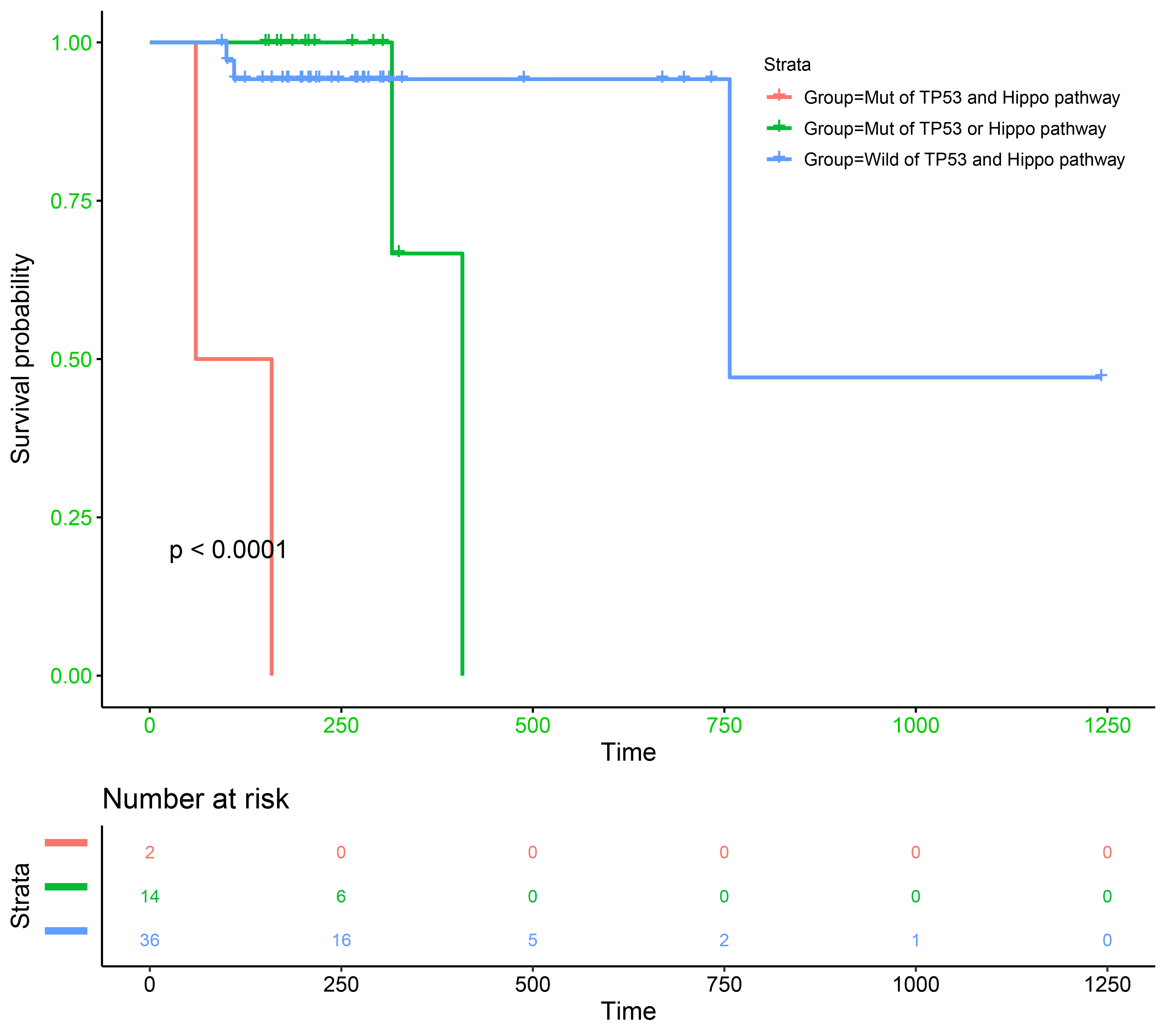

Supplement: Supplementary Table 1 — The overlapped mutated genes between the top 20 commonly mutated genes from COSMIC database and top 30 mutated genes our cohort. [file Image_3.tif]

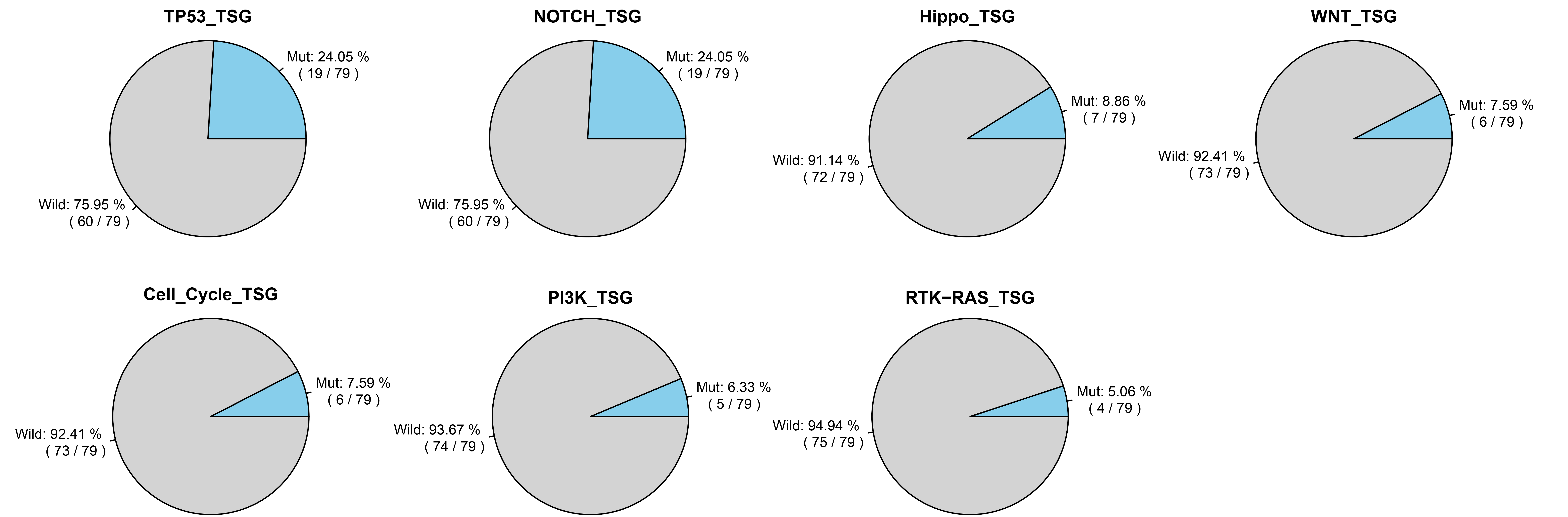

Supplement: Supplementary Figure 1 — Pie chart shows the mutation rates of seven canonical oncogenic signaling pathways (cell cycle, Hippo, Notch, PI3-Kinase, RTK-RAS, p53, and Wnt pathways) in 79 patients. [file Image_1.tif]

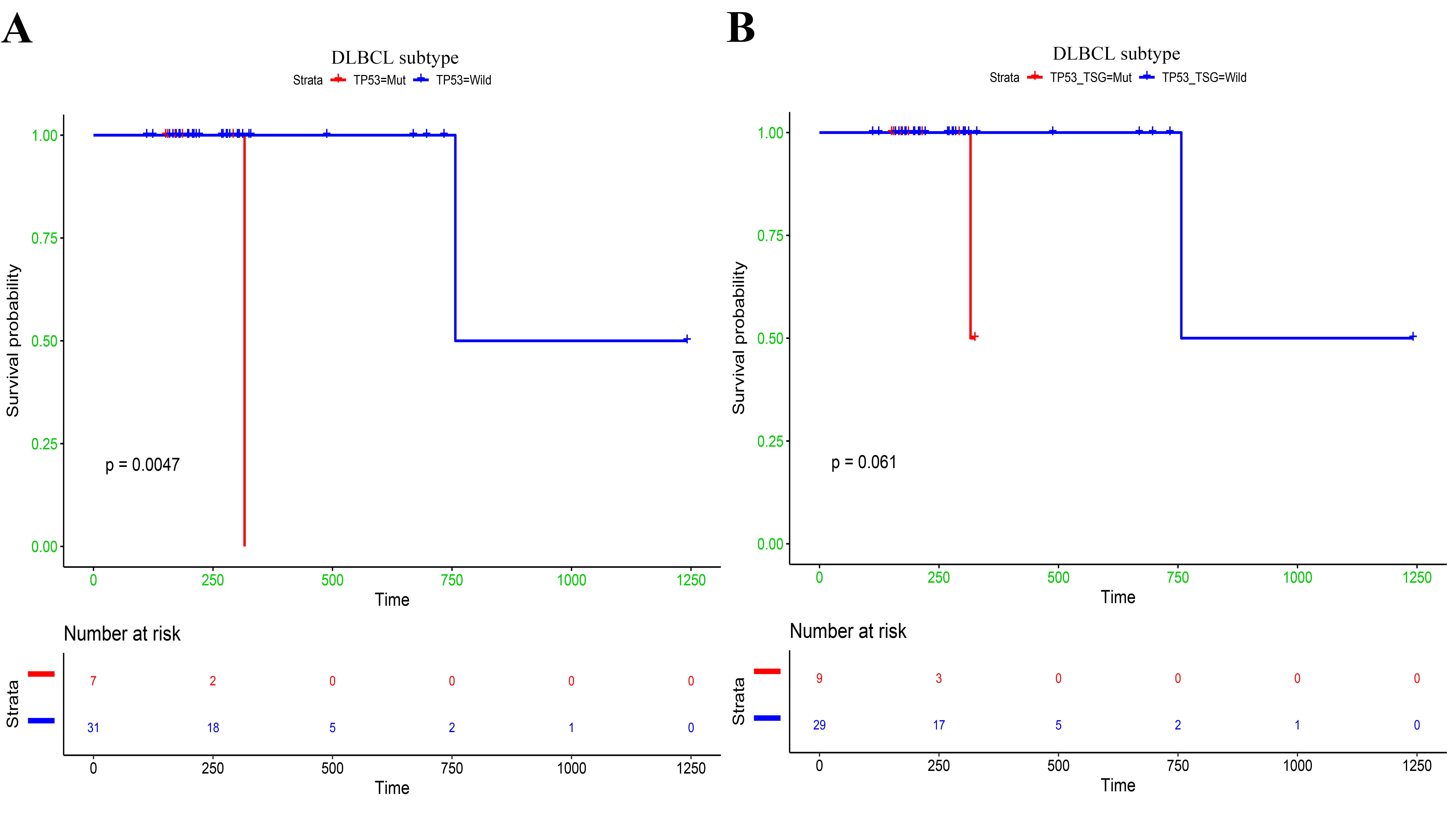

Supplement: Supplementary Figure 2 — Kaplan-Meier curves of PFS differences between the TP53-mutated and TP53 wild-type DLBCL patients (A) and between DLBCL patients with or without mutated TP53 pathway (B). [file Image_2.tif]
