# Supplementary material for: SEPT6_TRIM33 Gene Fusion and Mutated TP53 Pathway Associate With Unfavorable Prognosis in Patients With B-Cell Lymphomas
Source: Front Oncol. 2021 Dec 1;11:765544. doi: 10.3389/fonc.2021.765544 (PMC8671703; doi:10.3389/fonc.2021.765544)
Supplement: Supplementary Table 2 — Clinical characteristics of 52 patients for prognosis analysis. [file Table_1.docx]

**Table S1 The overlapped mutated genes between the top 20 commonly mutated genes from COSMIC database and the top 30 mutated genes in our cohort.**

| **Our cohort (Top 30)**  **(B-cell lymphomas, N=79)** | **COSMIC (Top20)**  **(DLBCL, N=413)** | | **Common genes** |
| --- | --- | --- | --- |
| TP53 (20%) | KMT2D (34 %) | | TP53 |
| MST1 (19%) | CREBBP (33 %) | | KMT2D |
| KMT2D (18%) | TP53 (31 %) | | MYD88 |
| GNAQ (16%) | BCL2 (25 %) | | CREBBP |
| MYD88 (15%) | PIM1 (19 %) | | ATM |
| DNMT3A (11%) | TNFRSF14 (17 %) | | BCL2 |
| GNAS (11%) | SOCS1 (15 %) | | PIM1 |
| PIM1 (11%) | EZH2 (13 %) | | CD79B |
| ARID1A (10%) | MYD88 (11 %) | | B2M |
| CREBBP (10%) | CARD11 (11 %) | |  |
| H3F3A (10%) | TET2 (11 %) | |  |
| KMT2C (9%) | TNFAIP3 (10 %) | |  |
| AR (8%) | B2M (10 %) | |  |
| ATM (8%) | FOXO1 (10 %) | |  |
| MEF2B (8%) | IRF4 (10 %) | |  |
| BTG2 (8%) | ATM (9 %) | |  |
| CCND3 (8%) | BRAF (6 %) | |  |
| CD70 (8%) | PTEN (5 %) | |  |
| CD79B (8%) | CD79B (3 %) | |  |
| CDKN2A (8%) | CD79A (3 %) | |  |
| GNA13 (8%) |  | |  |
| HLA−B (8%) |  | |  |
| NOTCH2 (8%) |  | |  |
| RANBP2 (8%) |  | |  |
| BTG1 (6%) |  | |  |
| FAT1 (6%) |  | |  |
| LRP1B (6%) |  | |  |
| B2M (5%) |  | |  |
| BCL2 (5%) |  |  | |
| HLA−A (5%) |  |  | |

Notes: COSMIC: The Catalogue Of Somatic Mutations In Cancer; DLBCL: Diffuse large B cell lymphoma.

**Table S2** Clinical characteristics of 52 patients for prognosis analysis

| **Variables** | **N (%)** |
| --- | --- |
| **Gender** |  |
| Male | 25 (48%) |
| Female | 27 (52%) |
| **Age, years, median (interquartile range)** | 56 (47-65) |
| **BMI (kg/m^2^)** |  |
| 18.5~23.9 | 33 (63.5%) |
| <18.5 | 5 (9.6%) |
| ≥24 | 13 (25.0%) |
| Unknown | 1 (1.9%) |
| **Smoking history** |  |
| Yes | 10 (19.2%) |
| No | 38 (73.1%) |
| Unknown | 4 (7.7%) |
| **Drinking history** |  |
| Yes | 16 (30.8%) |
| No | 36 (69.2%) |
| **Pathological diagnosis** |  |
| Diffuse large B-cell lymphoma | 38 (73.1%) |
| Follicular lymphoma | 4 (7.7%) |
| Marginal zone lymphoma | 1 (1.9%) |
| High-grade B-cell lymphoma | 1 (1.9%) |
| Large B cell lymphoma | 2 (3.9%) |
| Other* | 6 (11.5%) |
| **Stage** |  |
| I | 4 (7.7%) |
| II | 10 (19.2%) |
| III | 9 (17.3%) |
| IV | 26 (50.0%) |
| Unknown | 3 (5.8%) |
| **First-line therapy** |  |
| R-CHOP | 22 (42.3%) |
| CHOP | 1 (1.9%) |
| R-miniCHOP | 4 (7.7%) |
| R-COEP | 13 (25.0%) |
| R-DA-EPOCH | 3 (5.8%) |
| Other^#^ | 9 (13.5%) |
| **International Prognostic Index** |  |
| 0-1 | 18 (34.6%) |
| 2 | 13 (25.0%) |
| 3 | 11 (21.1%) |
| 4-5 | 7 (13.5%) |
| Unknown | 3 (5.8%) |
| **Response to treatment** |  |
| Complete response | 26 (50.0%) |
| Partial response | 19 (36.5%) |
| Stable disease | 2 (3.9%) |
| Progressive disease | 5 (9.6%) |

Notes: BMI: body mass index; R-CHOP: rituximab plus cyclophosphamide, doxorubicin, vincristine, and prednisone; R-miniCHOP: rituximab with low-does CHOP chemotherapy regimen; R-COEP: rituximab plus cyclophosphamide, vincristine, etoposide, and prednisone; R-DA-EPOCH: rituximab with dose-adjusted etoposide, prednisone, vincristine, cyclophosphamide, and doxorubicin.

Other* include subtypes of mantle cell lymphoma (MCL), B lymphoblastic lymphoma, [burkitt's](javascript:;) [lymphoma](javascript:;), lymphoplasmacytic lymphoma, lymphoblastic lymphoma and mature B-cell lymphomas.

Other^#^ include regimens of cyclophosphamide, epirubicin, vincristine, and prednisone (CEOP), rituximab combined with lenalidomide (R2)-CEOP (R2-CEOP), R2-gemox, rituximab combined with methotrexate, cytarabine and dexamethasone (R-MAD), hyperfractionated cyclophosphamide, vincristine, doxorubicin, and dexamethasone (Hyper-CVAD), rituximab plus methotrexate (R-MTX), R2-CHOP and R2-COEP.

**Table** **S3** Characteristics of subgroup patients with paired baseline and post-treatment samples (N=18)

| **Variables** | **N (%)** |
| --- | --- |
| **Gender** |  |
| Male | 6 (33.3%) |
| Female | 12 (66.7%) |
| **Age, years, median (interquartile range)** | 45.5 (57-61) |
| **Pathological diagnosis** |  |
| Diffuse large B-cell lymphoma | 15 (83.3%) |
| High-grade B-cell lymphoma | 1 (5.6%) |
| Follicular lymphoma | 1 (5.6%) |
| Mantle cell lymphoma | 1 (5.6%) |
| **Stage** |  |
| I | 1 (5.6%) |
| II | 3 (16.7%) |
| III | 3 (16.7%) |
| IV | 11 (61.1%) |
| **First-line therapy** |  |
| R-CHOP | 3 (16.7%) |
| R-COEP | 6 (33.3%) |
| R-DA-EPOCH  Other* | 1 (5.6%)  8 (44.4%) |
| **International Prognostic Index** |  |
| 0-1 | 8 (44.4%) |
| 2 | 3 (16.7%) |
| 3 | 4 (22.2%) |
| 4-5 | 3 (16.7%) |
| **Response to treatment** |  |
| Complete response | 6 (33.3%) |
| Partial response | 9 (50%) |
| Stable disease | 2 (11.1%) |
| Progressive disease | 1 (5.6%) |

Notes: R-CHOP: rituximab plus cyclophosphamide, doxorubicin, vincristine, and prednisone; R-COEP: rituximab plus cyclophosphamide, vincristine, etoposide, and prednisone; R-DA-EPOCH: rituximab with dose-adjusted etoposide, prednisone, vincristine, cyclophosphamide, and doxorubicin.

Other* include regimens of rituximab combined with methotrexate, cytarabine and dexamethasone (R-MAD), rituximab combined with lenalidomide and gemox (R2-Gemox), rituximab with low-does CHOP (R-miniCHOP).
